# Nutritional education during rehabilitation of children 6–24 months with acute malnutrition, under unavailability of therapeutic/supplementary foods: a retrospective study in rural Angola

**DOI:** 10.1186/s12887-021-02560-z

**Published:** 2021-02-24

**Authors:** Andrea Pietravalle, Martina Scilipoti, Francesco Cavallin, Magda Lonardi, Ivo Makonga Tshikamb, Claudia Robbiati, Daniele Trevisanuto, Giovanni Putoto

**Affiliations:** 1 Doctors with Africa CUAMM, Padua, Italy; 2 Doctors with Africa CUAMM, Chiulo, Angola; 3 Independent statistician, Solagna, Italy; 4 Missionary Catholic Hospital of Chiulo, Ombadja Municipality, Angola; 5 Department of Woman’s and Child’s Health, University of Padua, Padua, Italy

**Keywords:** Education, Nutritional rehabilitation, Acute malnutrition

## Abstract

**Background:**

Dietary counseling can play an important role in managing child malnutrition but is often inadequate or absent. Moreover, little emphasis is given to the usefulness of local available foods in the rehabilitation of malnourished children. This study aimed to evaluate the adherence and effectiveness of nutritional education during rehabilitation of children (6–24 months) with acute malnutrition, in a setting of unavailability of therapeutic/supplementary foods.

**Methods:**

Retrospective observational study on the adherence to dietary counseling and the impact on growth in children 6–24 months who were referred for acute malnutrition at the Catholic Mission Hospital of Chiulo (Angola) from August 2018 to January 2019. Main outcome measures were change in dietary habits and growth gain.

**Results:**

Sixty-four out of 120 children returned at first follow-up visit (default rate 47%). A change in dietary habits was reported in 32/64 (50%) children. Changing dietary habits was associated with an improved change in weight gain (MD 9.3 g/kg/day, 95%CI 4.2 to 14.3; *p* = 0.0005) and in weight/height ratio (MD 1.1 SD, 95%CI 0.7 to 1.4; *p* < 0.0001).

**Conclusions:**

A change in dietary habits after discharge was noted in only half of the patients who returned at first follow up visit, but it provided some advantages in term of weight gain and weight/height ratio. Further studies are needed to identify children at risk of low adherence to follow-up visits and low compliance to the nutritional recommendations, in order to increase the effectiveness of rehabilitation programs.

**Supplementary Information:**

The online version contains supplementary material available at 10.1186/s12887-021-02560-z.

## Introduction

In 2017, nearly 51 million of under-5 children were affected by acute malnutrition worldwide, with more than a quarter of them living in Africa [1]. The greatest risk of developing malnutrition occurs in the first 1000 days of life, from conception to 24 months of age [2]. Several factors concur to the incidence of malnutrition, including political and civil conflicts, environmental degradation, natural disasters, poverty, inadequate household access to food, infectious disease, inadequate breastfeeding and complementary feeding practices [3]. Malnutrition is the underlying cause in over 45% of all deaths among under-5 children [2]. Nonetheless, malnutrition is often associated with impaired growth and development, with adverse consequences in later life concerning health, intellectual ability, school achievement, work productivity, and earnings of survivors [2]. Ideally, the health-system infrastructure should integrate both prevention and treatment of malnutrition [4]. The most effective pathway to prevent malnutrition includes: adequate maternal nutrition before and during pregnancy and lactation; breastfeeding in the first 2 years of life; nutritive, diverse and safe foods in early childhood; healthy environment (i.e. access to basic health, water, hygiene and sanitation services); and opportunities for safe physical activity [5]. Ready-to-Use Therapeutic or Supplementary Foods (RUTF/RUSF) represent an effective and endorsed tool for the rehabilitation of children with severe and moderate acute malnutrition in both emergency and non-emergency settings, but the recurrent unavailability of these products is a frequent cause of nutrition program failure [6]. In addition, their long-term adverse effects should be taken into consideration [7]. Dietary counseling can play an important role in managing malnutrition thus should be an integral part of the treatment plan. However, dietary counseling is often inadequate or absent, and is often performed by health staff or volunteers with poor knowledge and communication skills [4]. Little emphasis is currently given to the usefulness of local available foods in the rehabilitation of malnourished children and improvements in counseling skills may help in conveying the most appropriate message [4]. This study aimed to evaluate the adherence to dietary counseling and the impact of changing dietary habits on growth, among children aged 6–24 months who were admitted with acute malnutrition at a rural district hospital, in a low-income setting where therapeutic and supplementary foods were lacking.

## Materials and methods

This is a retrospective observational study on the adherence to dietary counseling and the impact on growth in children who were referred for acute malnutrition at the Catholic Mission Hospital of Chiulo (Angola) from August 2018 to January 2019. The study was approved by the Ethics Committee of the Angolan Ministry of Health (ref. number 032020), which waived the need for written informed consent given the retrospective nature of the study and the use of anonymized data from hospital records. All methods were performed in accordance with the relevant guidelines and regulations.

### Setting

The Hospital of Catholic Mission of Chiulo is located in the province of Cunene (Angola). It is a district hospital implementing the Community Management of Acute Malnutrition (CMAM) program in a rural area of 12,263 km^2^ with 345,490 inhabitants (including 60,392 under-5 children) [8]. Chiulo Hospital is part of a network of 36 healthcare facilities involved in the national nutrition program and it works as a Stabilization Center (SC) for the inpatient care of malnourished children with complications, as well as an Outpatient Treatment Unit (OTU) for the rehabilitation phase after discharge. The nutritional rehabilitation unit counts 10 beds and is managed by a dedicated staff of doctors, nurses and paramedics. In 2018, 253 admissions were registered at the nutritional rehabilitation unit. In Angola, 90% of population living in rural areas belongs to the poorest social classes (54% to I quintile and 36% to II quintile) [8]. Education level is low among women aged 15–49 (35% attended only primary school and 22% received no education), while teenage childbearing involves 35% of adolescent women aged 15–19. Exclusive breastfeeding ranges from 62% among children aged 0–1 month to 17% among those aged 4–5 months, with a median duration of 3.1 months. Only 13% of children aged 6–24 months reach the WHO minimum acceptable diet standard. More than one-third (38%) of under-5 children are stunted while 5% are affected by acute malnutrition. Under-5 mortality rate differs by residence, province and household wealth, ranging from 68 deaths per 1000 live births in urban areas to 98 in rural areas.

### Community Management of Acute Malnutrition (CMAM) program

CMAM program identifies malnourished children at community level and refers them to SC or Outpatient Treatment Programs (OTP) according to the severity of malnutrition. Children with SAM/MAM without medical complications are treated in OTP, which provides routine medical treatment and nutrition rehabilitation with RUTF or RUSF for children with SAM or MAM respectively. Children attend outpatient care at regular intervals (every one or 2 weeks) until recovery is achieved (usually 2 months) [9]. Children with severe (SAM) or moderate (MAM) acute malnutrition and medical complications are admitted to SC until their clinical conditions are stabilized and complications resolved (usually four to 7 days). During this phase, F75 and F100 therapeutic milks are provided. Thereafter, children are usually treated in OTUs until nutritional recovery is achieved. Follow-up is performed in the OTUs, and relapsed children are referred again to the SC. At Chiulo Hospital, drugs, antibiotics, Resomal and F75-F100 therapeutic milk were properly offered, while the availability of therapeutic food was hampered by discontinued provision from the supply chain. A simple and prescriptive nutritional education, designed according to the WHO minimum acceptable diet standards [10] and using foods easily available in the study area, was provided before starting outpatient rehabilitation (detailed description in Supplementary Material: Table S1). Availability and affordability of the recommended foods were defined on the basis of the local health workers indications.

### Patients

All children aged 6–24 months with SAM/MAM discharged from SC were eligible for inclusion.

### Outcome measures

The outcome measures included the change in dietary habits and the weight change at first and second follow-up visits. The adherence to follow-up and the status (recovery, default, relapse, readmission) was also evaluated.

### Data collection

All data were retrospectively collected from hospital charts. Child characteristics included malnutrition status (MAM, SAM), age, sex, birth weight, duration of exclusive breastfeeding, comorbidities. Caregiver characteristics included age, education, number of pregnancies, and age of first pregnancy. Weight gain and status were retrieved at discharge and during follow-up. Dietary habits were recorded at admission and at each follow up visit. A “typical day” questionnaire was used to investigate the minimum meal frequency and the minimum dietary diversity (see section 2.6).

### Definitions

According to WHO classification [11], malnutrition was defined by the combination of clinical assessment and anthropometric measurements (Weight for Height ratio or Mid-Upper Arm Circumference). Weight for Height ratio < 3 Standard Deviation and Mid-Upper Arm Circumference ≤ 115 Millimeters indicated SAM, while values ≥3 and < 2 Standard Deviation or > 115 and < 124 Millimeters indicate MAM [11]. Dietary habits included minimum meal frequency and minimum dietary diversity [10]. Minimum meal frequency was defined as assuming daily solid/semi-solid foods at least 2 times for breastfed infants 6–8 months, 3 times for breastfed children 9–23 months, and 4 times for non-breastfed children 6–23 months. Minimum dietary diversity was defined as assuming at least 4 of the following 7 food groups: 1) grains, roots and tubers; 2) legumes and nuts; 3) dairy products; 4) flesh foods (meat, fish, poultry and liver/organ meats); 5) eggs; 6) vitamin-A rich fruits and vegetables; 7) other fruits and vegetables. Consumption of any amount of food from each food group was considered in the assessment. Achieving both minimum meal frequency and minimum dietary diversity was considered as change in dietary habits.

Weight change was evaluated in terms of weight-for-height ratio (expressed as standard deviations) and weight gain (expressed as g/kg/day) [11]. The rate of weight gain is used to monitor progress and effectiveness of rehabilitation phase. The WHO standards define the weight gain as poor (< 5 g/kg per day), moderate (5–10 g/kg per day) and good (> 10 g/kg per day) [11]. An admission represents the first contact with the program for treatment. WHO consider recovery when weight-for-height ratio is at least ≥ − 2 Standard Deviation or Mid-Upper-Arm Circumference is ≥125 mm and there is no edema for at least 2 weeks [11]. A default indicates a beneficiary who is absent for two consecutive weightings. A relapse is defined as a beneficiary readmitted to the program after having been successfully discharged as recovered within the last 2 months. A readmission identifies a beneficiary readmitted to the program within 2 months of leaving it for a reason other than recovery (e.g. defaulting or non-response) [9].

### Statistical analysis

The study included a convenient sample of all children who were referred for acute malnutrition at Chiulo Hospital (Angola) from August 2018 to January 2019. Categorical data were expressed as frequency (n) and percentage (%). Weight gain and weight/height ratio were expressed as mean and standard deviation (SD). The changes in weight gain and weight/height ratio during follow-up were compared among groups (according to change in dietary habits) using mixed-effect models. Effect sizes were reported as mean differences (MD) with 95% confidence intervals (CI). All tests were 2-sided and a *p*-value below 0.05 was considered statistically significant. Statistical analysis was performed using R 3.5 (R Foundation for Statistical Computing, Vienna, Austria) [12].

## Results

From August 2018 to January 2019, 139 children were admitted to the SC. Nineteen children (14%) died during hospitalization (13 of them died within 24 h from admission), while 120 (86%) were discharged and were included in the analysis. RUTF and RUSF were not available during the study period. Child and caregiver characteristics are reported in Table 1.

**Table 1:** Child and caregiver characteristics

	N of subjects	120
**Children**	**Moderate acute malnutrition (MAM)**	8 (7)
	**Severe acute malnutrition (SAM)**	112 (93)
	**Male:female**	56:64
	**Age:**	
	6–12 months	61 (51)
	13–24 months	59 (49)
	**Birth weight:**	
	< 2.5 kg (or defined as very small)	27 (22)
	≥2.5 kg	51 (42)
	Unknown	42 (35)
	**Duration of exclusive breastfeeding:**	
	< 1 month	3 (2)
	1–2 months	3 (2)
	3–6 months	66 (56)
	> 6 months	15 (12)
	Unknown	33 (28)
	**HIV**	6 (5)
	**Tuberculosis**	16 (13)
	**Dietary habits at admission:**	
	Get minimum meal frequency	5 (4)
	Get minimum dietary diversity	0 (0)
**Caregivers**	**Mother**	106 (88)
	**Others**	14 (12)
	**Age:**	
	≤ 14 years	0 (0)
	15–19 years	10 (8)
	20–30 years	50 (42)
	> 30 years	28 (23)
	Unknown	32 (27)
	**Educational status:**	
	Never attended school	76 (63)
	Primary school	19 (16)
	Secondary school	9 (8)
	Unknown	16 (13)
	**Number of pregnancies:**	
	< 3	31 (26)
	3–5	37 (31)
	> 5	31 (26)
	Unknown	21 (17)
	**Age of first pregnancy:**	
	< 15	22 (18)
	15–19	60 (50)
	20–30	10 (8)
	> 30	0 (0)
	Unknown	28 (24)

### Data expressed as n (%)

**Fig. 1:**
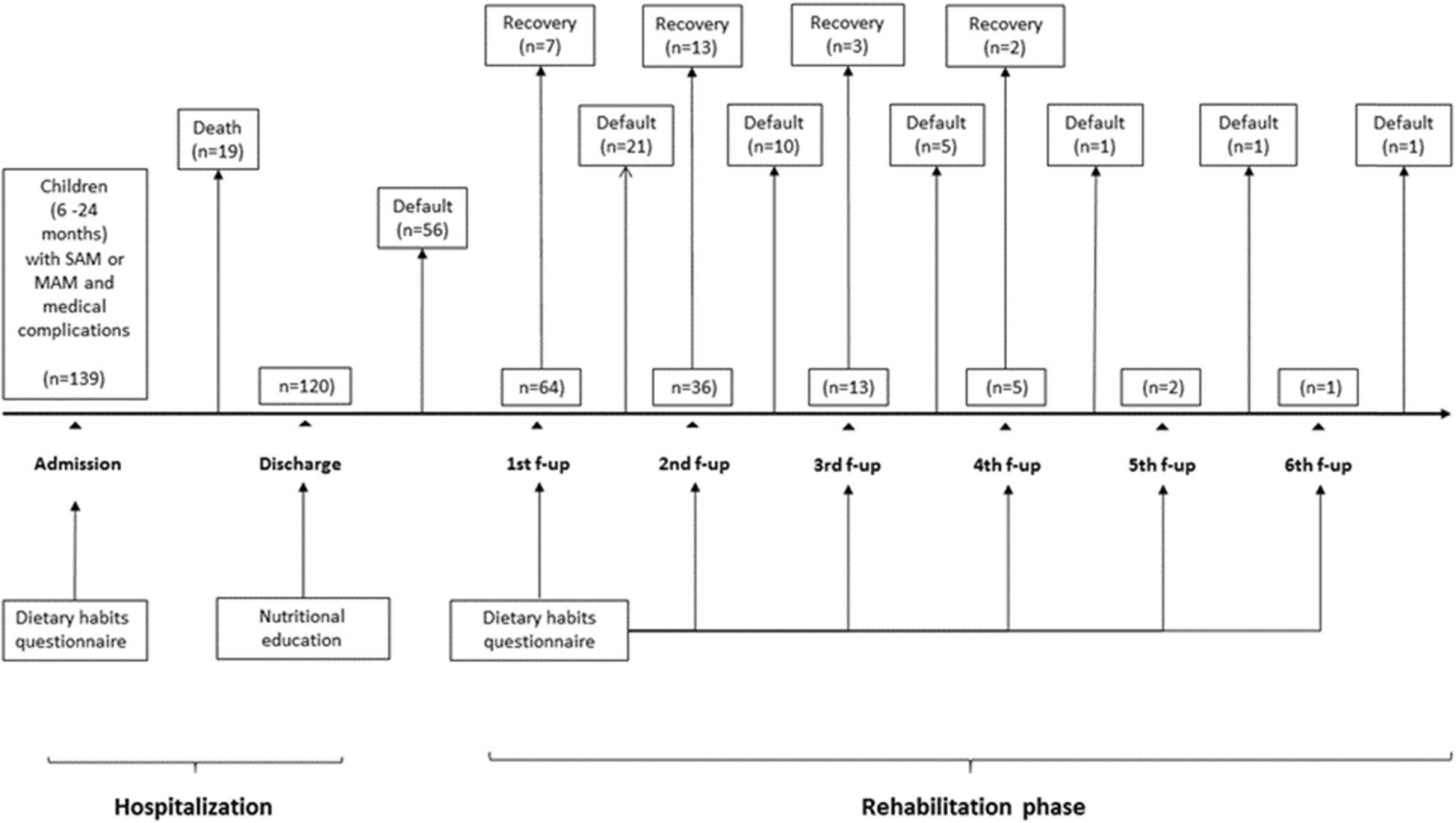
Study flow chart and scheme of intervention

At admission, eight children had MAM (7%) and 112 SAM (93%). The majority of caregivers were mothers (79%) and had not received formal education (67%). Fifty-six children (47%) were lost to follow-up after discharge, with a default rate of 78% during the follow-up period (Fig. 1). Lost to follow-up after discharge was not statistically associated with any child or caregiver characteristics (Supplementary Materials: Table S2). Only 26 children (22%) regularly attended the follow-up visits: 25 of them achieved recovery and one was readmitted for relapse (Fig. 1).

**Fig. 2:**
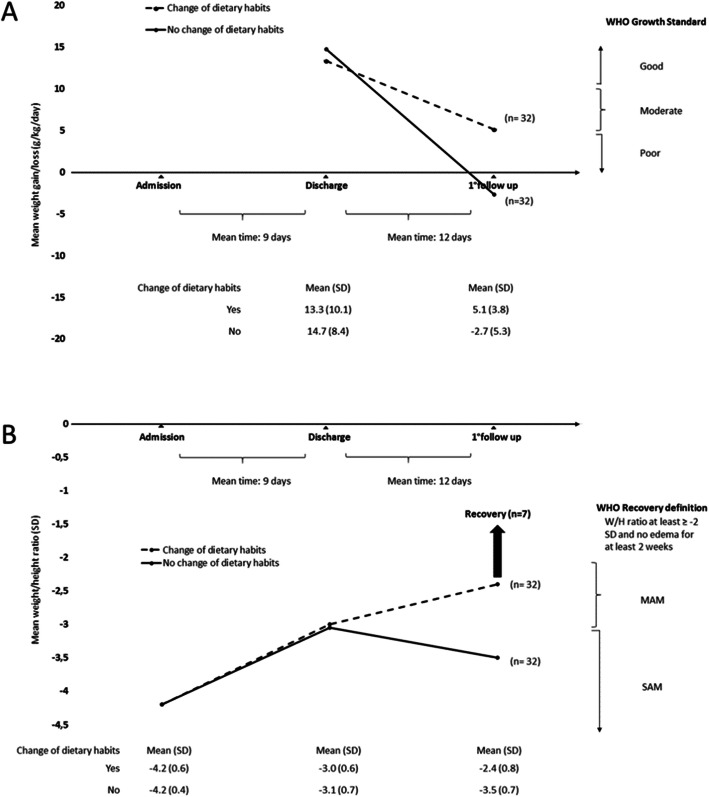
Mean weight gain/loss (**a**) and mean weight/height ratio (SD) (**b**) of 64 children who returned at first follow-up visit

**Fig. 3:**
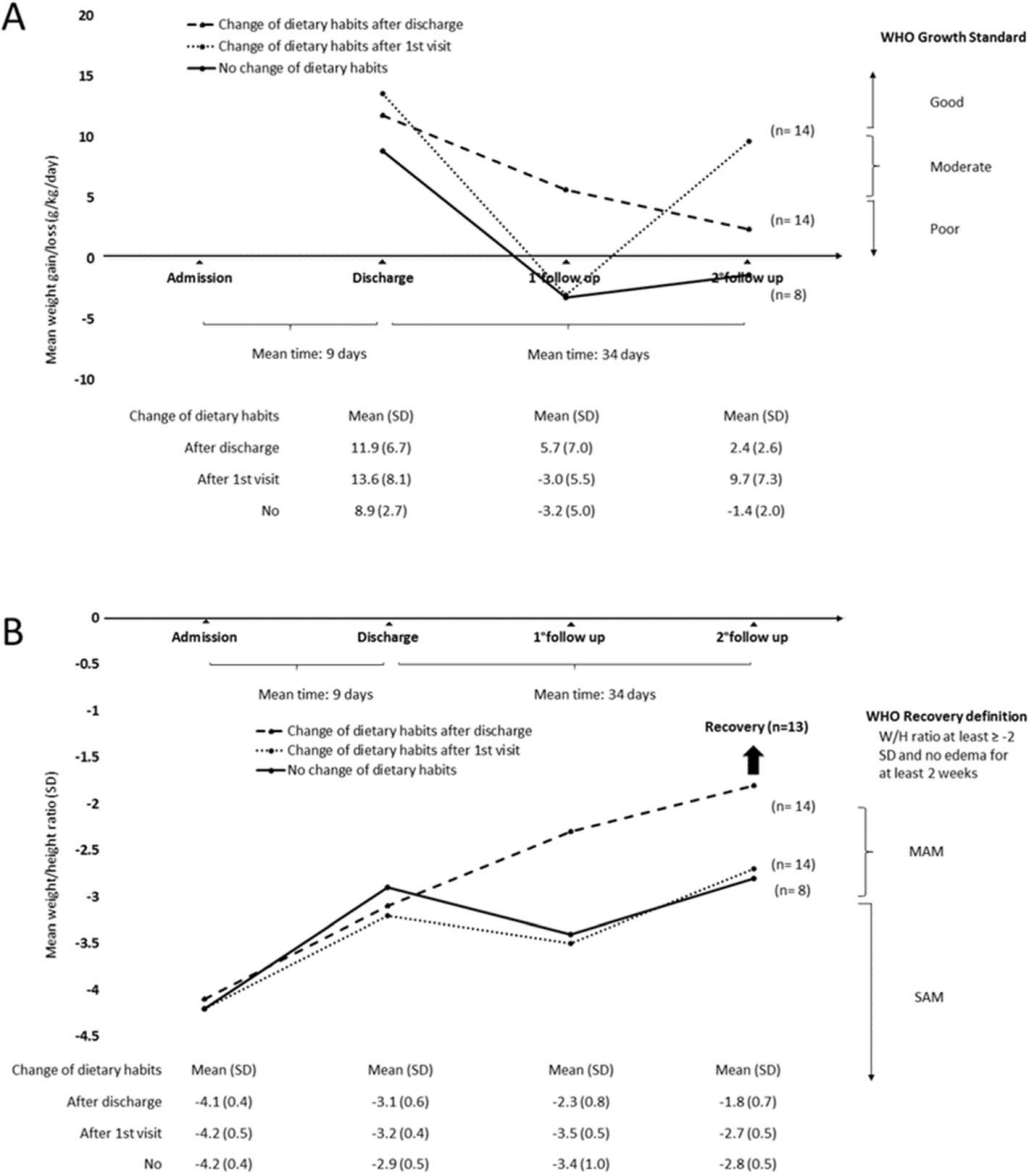
Mean weight gain/loss (**a**) and mean weight/height ratio (SD) (**b**) of 36 children who returned at second follow-up visit

At admission, none of the enrolled children was reaching the minimum acceptable diet standards. Among 64 children who returned at first follow-up visit, caregivers reported a change in dietary habits in 32 children (50%). Data on weight gain and weight/height ratio are shown in Fig. 2. Changing dietary habits was associated with an improved variation in weight gain (MD 9.3 g/kg/day, 95% CI 4.2 to 14.3; *p* = 0.0005) and in weight/height ratio (MD 1.1 SD, 95% CI 0.7 to 1.4; *p* < 0.0001). Seven children with changed dietary habits were considered recovered at the first visit. Among 36 children who returned at second follow-up visit, caregivers reported a change in dietary habits in 28 children (78%): 14 of them changed after discharge and 14 after the first follow-up visit. Data on weight gain and weight/height ratio are shown in Fig. 3.

In children who changed dietary habits after discharge, the variation in weight gain was not statistically different at first (MD 6.0 g/kg/day, 95% CI − 1.1 to 13.0; *p* = 0.11) or second (MD 0.8 g/kg/day, 95% CI − 6.3 to 7.9; *p* = 0.83) when compared to children who did not change dietary habits. However, they had improved change in weight/height ratio at first (MD 1.1 SD, 95% CI 0.5 to 1.6; *p* = 0.0004) and second (MD 1.0 SD, 95% CI 0.4 to 1.5; *p* = 0.001) follow-up visits. In children who changed dietary habits after first follow-up visit, the variation in weight gain was not statistically different at first (MD − 4.5 g/kg/day, 95% CI − 11.2 to 2.5; *p* = 0.23) or second (MD 6.4 g/kg/day, 95% CI − 0.7 to 13.5; *p* = 0.09) follow-up visits, when compared to children who did not change dietary habits. Their variation in weight/height ratio at first (MD 0.0 SD, 95% CI − 0.5 to 0.5; *p* = 0.90) and second (MD 0.1 SD, 95% CI − 0.4 to 0.7; *p* = 0.67) follow-up visits was also not statistically significant. Thirteen children with changed dietary habits were considered recovered at the second follow-up visit.

## Discussion

This study investigated the impact of a nutritional intervention involving children with SAM/MAM when RUTF and RUSF were unavailable. Half of the children were lost at the first follow-up visit, with an overall default rate of 78% during the follow-up period. A change in dietary habits after discharge was noted in only half of the patients who returned at first follow up visit, but it provided some advantages in term of weight gain and weight/height ratio.

Ready to use foods have proven effective at treating SAM and MAM in both emergency and non-emergency settings. However, their availability is often not guaranteed, and their limitations and potential impact on life course, health and nutrition should be taken into consideration [7]. Although effective in the short term, these products seem to have long-term adverse effects on preferences and eating habits, as well as on consumption patterns, thus increasing the risk of life-course exposure to the double burden of malnutrition (early undernutrition followed by later overweight) [13]. The absence of therapeutic foods usually leads to a complete paralysis of the CMAM program [14]. In such situation, OTUs are no longer able to provide nutritional rehabilitation at community level, and this prevents the SC from discharging patients, thus hindering the necessary turnover. The use of therapeutic foods can only be considered as a temporary remedy for the recovery of children from an acute state of malnutrition. Without a deep and sustained change in dietary habits, a durable improvement in health outcomes cannot be achieved. A change in dietary habits with the establishment of proper nutrition can be a sustainable, cost-effective and lasting strategy for prevention and treatment of undernutrition. In settings with locally available foodstuffs, nutritional education can improve feeding habits [15] and dietary diversity [16].

To our knowledge, available information on the effectiveness of family foods utilization in domiciliary rehabilitation of severe acute malnutrition is limited to two reports. In both of them, caregivers received nutritional education before discharge from SC, and multivitamins, iron [17] and zinc [18] were provided in addition to family foods. Their findings suggested that domiciliary administration of a proper diet, based on family foods, could effectively support catch-up growth during rehabilitation phase (with enhanced outcomes if micronutrient supplementation was provided). Our findings indicated a catch-up growth comparable with available report without zinc supplementation [17], thus strengthening the role of education on use of family food in domiciliary rehabilitation of severe acute malnutrition. Moreover, our data showed the association between growth gain and compliance to dietary recommendations. The caregivers only occasionally reported the possible inhibiting factors to practice the prescribed dietary behaviors. Although the information was not systematically collected, lack of affordability and lack of knowledge about food preparation were the most frequently reported factors. In our opinion, the enabling factors for the adoption of correct dietary behaviors, are to be found in the offered quality of care (in terms of time spent supporting and educating the caregivers) and in the recommendation of available and affordable food according to setting and seasonality. Of note, overall default rate was very high during follow-up period, with a relevant number of children who were lost at the first follow-up visit, thus confirming the role of default as the main cause of CMAM program failure [9]. Lack of community sensitization (i.e. awareness about the program and malnutrition), financial/opportunity costs (busy caregiver, distance to health facility, sick caregiver, lack of money) and low quality of care have been recognized as the main driving factors for default in malnutrition programs [6]. The high default rate can limit the impact of nutritional interventions for child malnutrition, as counseling during follow-up visits enhances adherence to nutritional indications and plays an important role in successful rehabilitation programs [4].

This study has some limitations that should be considered. First, the retrospective observational design limited quality and availability of data. Second, the low sample size and the high default rate restricted data analysis. Third, information about children not attending follow-up visits was lacking.

These limitations could be overcome by planning prospective studies with pre-established data collection forms, enhanced communication on importance of adherence to follow-up visits and retrieval of information on children not compliant to follow-up visits. Further studies are also needed to identify children at risk of low adherence to follow-up visits and low compliance to the nutritional recommendations, in order to increase the effectiveness of rehabilitation programs.

Our findings support the hypothesis that family foods can play an important role in the rehabilitation phase of children with acute malnutrition. Adequate dietary counseling should be implemented as part of CMAM programs, in order to enhance the impact of the rehabilitation intervention. However, the suboptimal proportion of children with changed dietary habits suggests the need for strategies to ensure full adherence to the counseling. Effective approaches may include the frequent, regular exposure to a few simple, uniform, age-appropriate and prescriptive messages, and the involvement of the community for social support (i.e. peer support groups and shared experience) [4, 19]. In addition, the high default rate during follow-up period calls for appropriate actions to increase adherence to follow-up visits. Building a strong relationship between health care staff and child caregivers, and fostering active participation of the community can play an important role in strengthening CMAM implementation.

## Conclusions

In this nutritional program, half of the children were lost at the first follow-up visit. A change in dietary habits after discharge was noted in only half of the children who returned at first follow up visit, but it provided some advantages in term of weight gain and weight/height ratio. Further studies are needed to identify children at risk of low adherence to follow-up visits and low compliance to the nutritional recommendations, in order to increase the effectiveness of rehabilitation programs.

## Supplementary Information

**Additional file 1: Table S1.** “Description of the prescriptive nutritional education provided by health caregivers to child caregivers at Chiulo Hospital before starting outpatient rehabilitation.”

**Additional file 2: Table S2.** "Comparison of child and caregiver characteristics between children who attended first follow-up visit and those who were lost to follow-up after discharge."

## Data Availability

The datasets used and/or analysed during the current study are available from the corresponding author on reasonable request.

## Abbreviations

RUTF/RUSF: Ready-to-Use Therapeutic /Supplementary Foods
CMAM: Community Management of Acute Malnutrition
SAM: Severe Acute Malnutrition
MAM: Moderate Acute Malnutrition
SC: Stabilization Center
OTP: Outpatient Treatment Program
OTU: Outpatient Treatment Unit
