# Supplementary material for: Nutritional education during rehabilitation of children 6–24 months with acute malnutrition, under unavailability of therapeutic/supplementary foods: a retrospective study in rural Angola
Source: BMC Pediatr. 2021 Feb 24;21:94. doi: 10.1186/s12887-021-02560-z (PMC7903716; doi:10.1186/s12887-021-02560-z)
Supplement: Supplementary file 1 — Additional file 1: Table S1. “Description of the prescriptive nutritional education provided by health caregivers to child caregivers at Chiulo Hospital before starting outpatient rehabilitation.” [file 12887_2021_2560_MOESM1_ESM.docx]

SUPPLEMENTARY MATERIAL: Table S1

Description of the prescriptive nutritional education provided by health caregivers to child caregivers at Chiulo Hospital before starting outpatient rehabilitation.

| Topic | Specific topic | Implementation |
| --- | --- | --- |
| Hygiene | Food hygiene | Health care givers advised child caregivers to wash the food thoroughly and boil water used for cooking; to use soap (if available) for washing the dishes; to not use feeding bottles, as more difficult to sanitize. Health care givers provided soap when available. |
|  | Hand hygiene | Health care givers advised child caregivers to wash their hands before and after preparing food, and before eating; to use soap, if available. Health care givers provided soap when available. |
|  | Drinking-water source and treatment | Health care givers advised child caregivers to not use unimproved drinking water sources, such as unprotected dug well, unprotected spring, cart with small tank/drum, tanker truck, and surface water (river, dam, lake, pond, stream, canal, irrigation channels). Health care givers provided water disinfectant when available. |
| Diet | Food groups and dietary diversity | Health care givers explained food groups and the importance of dietary diversity to child caregivers. Minimum dietary diversity was defined as assuming at least 4 of the following 7 food groups: 1) grains, roots and tubers; 2) legumes and nuts; 3) dairy products; 4) flesh foods (meat, fish, poultry and liver/organ meats); 5) eggs; 6) vitamin-A rich fruits and vegetables; 7) other fruits and vegetables. |
|  | Food frequency | Health care givers explained the importance of adequate food frequency to child caregivers. Minimum meal frequency was defined as assuming daily solid/semi-solid foods at least 2 times for breastfed infants 6–8 months, 3 times for breastfed children 9–23 months, and 4 times for non-breastfed children 6–23 months. |
| Health | Prevention of infections and diseases | Health care givers provided general education to child caregivers on the importance of hygiene, prevention of infections and diseases |
| Recipes using locally available food | Recipe 1 | Ingredients: beans (2 tablespoons, 60 gr), 1 potato, 1 carrot, rice (2 tablespoons, 60 gr), corn meal (2 tablespoons, 30 gr). Cooking ingredients in hot water and adding oil and salt. |
|  | Recipe 2 | Ingredients: 1 potato, 1 carrot, 1 onion, rice or pasta (2 tablespoons, 30 gr), 1 egg. Cooking vegetables and rise/pasta in hot water separately, then mixing together adding the egg, oil and salt. |
|  | Recipe 3 | Ingredients: meat (cow, pig, or chicken) or fish, 1 potato, 1 carrot, 1 onion, rice (2 tablespoons, 30 gr). Cooking rice with vegetables and meat/fish separately, then mixing together as porridge and adding oil. |

The education was performed daily for the entire period of the in-hospital rehabilitation phase, involving all caregivers, regardless of the time of discharge. Cooking demonstrations were performed two or three times a week using the recipes described below. Nutritional education was repeated at each follow-up visit, also involving the caregivers of the children were still hospitalized.
