# Supplementary material for: Nutritional education during rehabilitation of children 6–24 months with acute malnutrition, under unavailability of therapeutic/supplementary foods: a retrospective study in rural Angola
Source: BMC Pediatr. 2021 Feb 24;21:94. doi: 10.1186/s12887-021-02560-z (PMC7903716; doi:10.1186/s12887-021-02560-z)
Supplement: Supplementary file 2 — Additional file 2: Table S2. "Comparison of child and caregiver characteristics between children who attended first follow-up visit and those who were lost to follow-up after discharge." [file 12887_2021_2560_MOESM2_ESM.docx]

**Supplementary Materials: Table S2:** Comparison of child and caregiver characteristics between children who attended first follow-up visit and those who were lost to follow-up after discharge.

|  |  | Children who attended first follow-up visit | Children who were lost to follow-up after discharge | p-value |
| --- | --- | --- | --- | --- |
|  | N of subjects | 64 | 56 | - |
| Children | Moderate acute malnutrition (MAM)  Severe acute malnutrition (SAM) | 2 (3)  62 (97) | 6 (11)  50 (89) | 0.20 |
|  | Male:female | 26:38 | 30:26 | 0.22 |
|  | Age:  6-12 months  13-24 months | 32 (50)  32 (50) | 29 (52)  27 (48) | 0.99 |
|  | Birth weight: ^a^  < 2.5 kg (or defined as very small)  ≥2.5 kg | 15 (32)  31 (68) | 12 (37)  20 (63) | 0.84 |
|  | Duration of exclusive breastfeeding: ^b^  <1 month  1-2 months  3-6 months  >6 months | 2 (4)  0 (0)  43 (81)  8 (15) | 1 (3)  3 (9)  23 (68)  7 (20) | 0.14 |
|  | HIV | 5 (8) | 1 (2) | 0.21 |
|  | Tuberculosis | 9 (14) | 7 (13) | 0.99 |
|  | Dietary habits at admission:  Get minimum meal frequency  Get minimum dietary diversity | 3 (5)  0 (0) | 2 (4)  0(0) | 0.99  NA |
| Caregivers | Mother  Others | 57 (89)  7 (11) | 49 (87)  7 (13) | 0.99 |
|  | Age: ^c^  ≤ 14 years  15-19 years  20-30 years  >30 years | 0 (0)  5 (9)  32 (57)  19 (34) | 0 (0)  5 (16)  18 (56)  9 (28) | 0.63 |
|  | Educational status: ^d^  Never attended school  Primary school  Secondary school | 43 (72)  12 (20)  5 (8) | 33 (75)  7 (16)  4 (9) | 0.94 |
|  | Number of pregnancies: ^e^  <3  3-5  >5 | 16 (29)  20 (36)  19 (35) | 15 (34)  17 (39)  12 (27) | 0.72 |
|  | Age of first pregnancy: ^f^  <15  15-19  20-30  >30 | 11 (20)  40 (73)  4 (7)  0 (0) | 11 (30)  20 (54)  6 (16)  0 (0) | 0.17 |

Data expressed as n (%). Data not available in ^a^42, ^b^33, ^c^32, ^d^16, ^e^21 and ^f^28 patients.
